# Sex moderations in the relationship between aortic stiffness, cognition, and cerebrovascular reactivity in healthy older adults

**DOI:** 10.1371/journal.pone.0257815

**Published:** 2021-09-28

**Authors:** Dalia Sabra, Brittany Intzandt, Laurence Desjardins-Crepeau, Antoine Langeard, Christopher J. Steele, Frédérique Frouin, Richard D. Hoge, Louis Bherer, Claudine J. Gauthier

**Affiliations:** 1 Faculty of Medicine, Department of Biomedical Science, Université de Montreal, Montreal, QC, Canada; 2 Research Center, Montreal Heart Institute, Montreal, QC, Canada; 3 Centre de recherche de l’Institut Universitaire de Gériatrie de Montréal (CRIUGM), Montréal, QC, Canada; 4 Department of Medicine, Universite de Montreal, Montreal, QC, Canada; 5 PERFORM Centre, Concordia University, Montreal, QC, Canada; 6 INDI Department, Concordia University, Montreal, QC, Canada; 7 Department of Neurology, Max Planck Institute for Human Cognitive and Brain Sciences, Leipzig, Germany; 8 Department of Psychology, Concordia University, Montreal, QC, Canada; 9 LITO laboratory, Inserm, Institut Curie, Orsay, France; 10 Montreal Neurological Institute, Montreal, QC, Canada; 11 Department of Neurology and Neurosurgery, McGill University, Montreal, QC, Canada; 12 Physics Department, Concordia University, Montreal, QC, Canada; University of Texas at Dallas, UNITED STATES

## Abstract

It is well established that sex differences exist in the manifestation of vascular diseases. Arterial stiffness (AS) has been associated with changes in cerebrovascular reactivity (CVR) and cognitive decline in aging. Specifically, older adults with increased AS show a decline on executive function (EF) tasks. Interestingly, the relationship between AS and CVR is more complex, where some studies show decreased CVR with increased AS, and others demonstrate preserved CVR despite higher AS. Here, we investigated the possible role of sex on these hemodynamic relationships. Acquisitions were completed in 48 older adults. Pseudo-continuous arterial spin labeling (pCASL) data were collected during a hypercapnia challenge. Aortic pulse wave velocity (PWV) data was acquired using cine phase contrast velocity series. Cognitive function was assessed with a comprehensive neuropsychological battery, and a composite score for EF was calculated using four cognitive tests from the neuropsychological battery. A moderation model test revealed that sex moderated the relationship between PWV and CVR and PWV and EF, but not between CVR and EF. Together, our results indicate that the relationships between central stiffness, cerebral hemodynamics and cognition are in part mediated by sex.

## 1. Introduction

Cardiovascular diseases (CVD) are the leading cause of death worldwide in males and females, and on average, someone dies of CVD every 38 seconds, resulting in 2,303 deaths per day due to CVDs [1]. Cardiovascular risk is highly sex-dependent, as males exhibit greater incidences and prevalence than females [2]. However, the risk of heart disease is often underestimated in females. This could be explained, in part, by the findings of epidemiological studies indicating that premenopausal females are relatively protected from CVDs when compared to age-matched males [3–5]. Yet, the incidence of CVD increases disproportionately in females 7–10 years after menopause [3–5], and is the most common cause of death in females over the age of 65 years [6]. This highlights the need to better understand the underlying mechanisms of sex differences in the development of cardiovascular diseases. The pathophysiology underlying CVDs is thought to differ depending on the presence of sex hormones leading to differences in vascular properties, including differences in vascular tone [5]. Notably, estrogen is thought to have a positive effect on the inner layer of the artery wall, helping to keep blood vessels flexible, and allowing them to relax and expand to accommodate increases in blood flow [6,7]. Consequently, a decline in estrogen in postmenopausal females may lead to arterial stiffening (AS) and thus contribute to the increased prevalence of CVD in females in later life [8–10]. Moreover, recent work suggests a potential protective role of testosterone against AS. Specifically, low testosterone levels in males is associated with greater AS [11] and augmentation index, an indirect measure of carotid stiffness [12] It is well established that elevated AS is an independent predictor of CVD [13] The elasticity of large arteries allows for the dampening of the arterial pressure waveform, transforming the pulsatile flow at the heart level into steady blood flow into the micro-vessels [14–17]. Unfortunately, during aging, large arteries (e.g., the aorta, the carotids etc.) become stiffer and show a reduced capacity to dampen the arterial pressure waveform [16,17]. With aging, the elastic properties of blood vessel walls are known to deteriorate [18]. In particular, the ratio between elastin and collagen changes in favor of collagen, making vessels stiffer [18]. Considering the impact of AS on vascular health, non-invasive methods have been developed to measure it, among which pulse wave velocity (PWV) of the aorta is considered to be the gold standard [19]. The impact of AS differs considerably between males and females because of numerous endogenous factors, such as the previously mentioned sex hormones, and biochemical properties of the arteries [20,21]. For instance, it has been shown that aged male and female monkeys develop similar levels of AS but a decrease in elastin was noted only in male monkeys [22]. In addition, dyslipidemia and glucose contribute to a modest increase in arterial stiffness only in females [23]. Moreover, it has been shown that the association between AS and mortality is almost two-fold higher in females compared to males after menopause [24], likely showing differences in pathophysiological mechanisms.

AS is also associated with downstream organ damage, especially in high-flow organs such as the brain [13,15,17,25,26]. Indeed, high pulsatile flow following AS may damage cerebral micro vessels, thus, leading progressively to changes in cerebral blood flow (CBF) [27–29]. Increased stiffness is also associated with changes in cerebrovascular reactivity (CVR), defined as the ability of micro vessels to increase blood flow in response to a vasodilatory stimulus [30,31]. It can be hypothesized that increased stiffening of the aorta and higher pulse pressure may lead to damage in downstream vessels from the damaging effects of the pressure amplification caused by large artery stiffness. Therefore, greater artery stiffness may contribute to the impaired ability of the cerebrovascular to dilate maximally to augment cerebral blood flow (CBF) in older adults. Yet, the literature has found conflicting results, where some have reported reductions in CVR among older adults with greater aortic stiffness using positron-emission tomography (PET) and transcranial doppler (TCD) [30,32], while others demonstrate preserved CVR in the presence of higher aortic PWV [31,33] using arterial spin labeling (ASL), an MRI technique for noninvasive quantification of CBF. Because these imaging modalities (TCD, PET, ASL) are sensitive to CVR arising from different vessel sizes, these results indicate that the relationship between PWV and CVR is complex. With the evidence that sex differences exist in the manifestation of AS, it is also unclear if the conflicting results could partly be due to sex-specific characteristics.

Another consequence of a stiffer vascular network is a change in cognitive function [34]. There is accumulating evidence from cross-sectional studies that AS is associated with the pathogenesis of cognitive decline in both males and females [34–36] in age-sensitive domains such as processing speed (PS) and executive functions (EF) [37,38]. Interestingly, associations between PWV, impaired CVR and severity of dementia have also been established [39]. Indeed, reduced reactivity has been linked to decreased executive functioning [40]. Furthermore, evidence for sex differences has been reported in several specific cognitive domains [41] where males display stronger associations between executive functioning and hemodynamic measurements compared to females [42] Yet, no study to date has investigated the role of sex on the relationship between cognition and neuroimaging markers of brain hemodynamics. Thus, the purpose of this study is to clarify the impact of sex-related differences on the link between PWV, cognitive performance and CVR. We hypothesize that this study will detect sex differences, with males displaying stronger associations

## 2. Methods

### 2.1 Participants

Fifty-four healthy older adults (17 males, mean age 63 ± 5 years) completed a magnetic resonance imaging (MRI) session. Participants were recruited through a participant data base at the Centre de recherche de l’Institut universitaire de gériatrie de Montréal and from Laboratoire D’Étude de la Santé cognitive des Ainés. Inclusion criteria for participation included being in the age range of 55 to 75 years, approval by a geriatrician to participate, non-smoker, no evidence of cognitive impairment as determined through cognitive tests conducted by a neuropsychologist, and MRI compatibility. In addition, the Mini Mental Status Examination (MMSE) was administered, a global cognitive screening tool used for dementia. A score of less than 26 (out of 30) was used as an additional exclusion criterion [43,44] (no participant was excluded from the sample for this reason). Other exclusion criteria included individuals on hormone replacement therapy or taking prescription medication known to be vasoactive (e.g., anti-hypertensive drugs, statins, etc.), presence of cardiac disease, hypertension (including use of anti-hypertensive medication), neurological or psychiatric illnesses, diabetes, asthma, thyroid disorders, smoking within the last 5 years, or excessive drinking (more than two drinks per day). All procedures were approved by Comité mixte d’éthique de la recherche du Regroupement Neuroimagerie/Québec and were conducted according to the Declaration of Helsinki. All participants provided written informed consent. From all participants that were recruited, a total of 48 older adults (17 males, mean age 63 ± 5 years) were included in the analysis. The six participants (2 females) were excluded as outliers since they were more than 2.5 standard deviations above or below the mean of PWV values which is our primary index of vascular health. Exclusion of these participants did not change the results (see S1–S3 Figs). All participants first underwent a brain MRI scan with a hypercapnic manipulation followed by an aortic exam to measure central stiffness.

### 2.2 Cognitive functioning

Cognitive function was assessed with a comprehensive neuropsychological battery consisting of the following cognitive tests: Similarities, Digit Span Backwards, Digit Span forward, Digit Symbol, Color-word interference test (CWIT), and Trail Making Tests, parts A and B [45,46]. A composite score for executive function (EF) was calculated using four cognitive tests from the neuropsychological battery that included the CWIT Inhibition and switching conditions, Trail making test part B and the digit span backward. The trail making test part B (TMT-B), the CWIT inhibition condition and the CWIT switching condition were timed in seconds where a low score (faster response) indicates better functioning. The digit span backward was calculated as the number of successful trials where a higher score indicates better EF.

Individual raw scores for each test were transformed into z-scores. The scores that were response time were multiplied by -1 so that a higher EF composite score indicates better cognitive functioning. Cronbach’s alpha was used as a test of reliability to look at the internal consistency for the group of variables. A Cronbach alpha of 0.789 was computed for executive function showing good internal consistency.

In addition, a composite score for processing speed was also calculated using four cognitive tests from the neuropsychological battery. This score included the trail making test part A, the Stroop color naming condition, the Stroop reading condition and substitution. A Cronbach alpha of 0.716 was computed for processing speed, showing inadequate internal consistency, and was not included for further analysis.

### 2.3 Hypercapnia

As previously described [44–46], the hypercapnic manipulation was completed with a computer-controlled gas system with a sequential gas delivery circuit (RespirAct™, Thornhill Research Inc., Toronto, Canada). The hypercapnic manipulation consisted of two, 2-minute blocks of hypercapnia, with 2 minutes of air before and after each hypercapnia block. End-tidal partial pressure of CO_2_ (ETCO2) was targeted at 40 mmHg at baseline and 45 mmHg during the hypercapnia blocks. End-tidal partial pressure of O_2_ (ETO2) was targeted to be 100 mmHg throughout the experiment. The RespirAct apparatus works through prospective targeting using measured metabolic values, the amount of CO_2_ provided is personalized to fit each individual’s metabolic rate. The system adjusts the CO_2_ concentration provided over time, to take into account CO_2_ dissolved in blood and tissue as CO_2_ is provided over longer periods of time. Previous work has used this level of CO_2_ to obtain robust CBF changes around 40% [44–46]. One of the advantages of this system is that EtO2 are also targeted, ensuring that hypercapnia is not associated with O_2_-induced vasoconstriction from concomitant hyperoxia, as fixed inspired challenges are liable to. Participants breathed through a soft plastic mask that was firmly placed on their face with adhesive tape (Tegaderm 3M Healthcare, St. Paul MN) to ensure that no leaks were present. Participants completed the breathing manipulation once prior to being in the scanner to ensure adequate comfort levels.

### 2.4 Brain MRI acquisition

All acquisitions were completed on a Siemens TIM Trio 3T MRI system (Siemens Medical Solutions, Erlangen, Germany). A 32-channel vendor-supplied head coil was used for all acquisitions. An anatomical 1 mm^3^ MPRAGE acquisition (TR/TE/flip angle = 300 ms/3 ms/90°, 256x240 matrix) was acquired for the registration process from native to standard space, and to measure grey matter partial volume. A fluid attenuation inversion recovery (FLAIR) acquisition with the parameters: TR/TE/flip angle 9000 ms/107 ms/120°, an inversion time of 2500 ms, 512 x 512 matrix for an in-plane resolution of 0.43 x 0.43 mm and 25 slices of 4.8 mm was used to estimate the presence and severity of white-matter hyperintensities. In addition, a pseudo-continuous arterial spin labeling (pCASL) acquisition was acquired, providing simultaneous BOLD contrast using dual-echo readouts (TR/TE1/TE2/flip angle = 2000 ms/10 ms/30 ms/90°) with 4x4x7 mm^3^ voxels, 64 x 64 matrix and 11 slices, post-label delay = 900 ms, tag duration = 1.5 s, and a 100 mm gap during a hypercapnia challenge (5 mmHg end-tidal CO2 change, iso-oxic during two, 2 min blocks).

### 2.5 Data analysis

Preprocessing of TI-weighted MPRAGE images were done using voxel-based morphometry (VBM) in SPM’s Computational Anatomy Toolbox (CAT) 12 [47–49] to segment grey matter, white matter, and cerebrospinal fluid (CSF). The registration matrix from T1 space to MNI space was calculated as part of the VBM pipeline. Co-registration of native CVR data was done using a non-linear rigid registration with ANTS [50] with a b-spline interpolation to bring them from native to individual T1 space. CAT12 was then used to register from T1 to standard space using a Gaussian smoothing kernel of 8 mm and a non-linear registration with 12 degrees of freedom as previously described [46].

### 2.6 Resting CBF analysis

Resting CBF was quantified using the first echo of the whole pCASL data time series. Resting CBF was calculated as previously described [46]. The average of the control images was used for each participant with modeling of the T1-recovery to obtain the fully recovered magnetization (M0) using AFNI, FSL and in-house scripts. CSF masks were created individually for each older adult to use as a CSF M0 for CBF quantification. 10 voxels were manually chosen in the same axial slice for each participant, within the posterior lateral ventricles. All individual masks were visually inspected to ensure the ROIs were in the ventricles. The M0 was then estimated from the control time series and estimated using a monoexponentially recovery with a T1 value of 1.65s. Due to varying anatomical structures, each CSF mask was visually inspected to ensure that the region of interest was entirely located in the ventricles. The Bayesian inference for arterial spin labeling MRI toolbox (BASIL) was used for CBF quantification with the following parameters: labeling: cASL/pcASL; bolus duration: constant (1.5s), post label delay: 0.9s; calibration image: average of the control images; reference tissue type: CSF; mask: CSF mask for each participant; CSF TI: 4.3 s; TE:10 ms; T2: 750 ms; blood T2: 150 ms; arterial transit time: 1.3 s, T1: 1.3s, TI blood: 1.65 s, inversion efficiency: 0.85 [46].

### 2.7 CVR analysis

CBF-CVR was processed using Neurolens2 [45,46]. Preprocessing of all raw images included motion correction and spatial smoothing using a 6 mm Gaussian kernel. Volumes with movement greater than 1mm translation and 1.5-degree rotation were removed from the time series and motion parameters were regressed out in the GLM used to quantify perfusion over time. The data was also scrubbed by removing any volume with artifacts, such as volumes with large negative areas not following anatomical boundaries. A partial volume correction was also performed to minimize the effects of WM and CSF on CBF values. The CBF signal was isolated from the first series of echoes using linear surround subtraction [46,51–53]. The CBF fractional change during hypercapnia was obtained by fitting a general linear model to the CBF signal and dividing the estimated effect size by the estimated constant term. Glover’s parameters (1999) [54] for a single-gamma hemodynamic response function were used when fitting the linear models, which included linear, quadratic, and third order polynomials representing baseline signal and drifts. The CBF percent change obtained was then divided by the average end-tidal CO2 change during the hypercapnia manipulation for each participant to yield CBF-CVR. The baseline CBF was then used to compute absolute values of CBF-CVR. For visualization of data quality and example maps, see [44,46].

### 2.8 Vascular lesion quantification

White matter hyperintensity volume (WMH) for the whole brain was quantified semi-automatically. As previously described [44,46] visual identification on FLAIR images were completed by a single rater who was blinded to clinical information, which were then delineated using the Jim image analysis package, version 6.0 (Xinapse Systems Ltd, Northants, UK) by an expert rater. Even though this sample is healthy given the age range, WMH volume varied widely within the sample. Given that some participants had large WMH volumes, and some had none, and given that WMH are thought to reflect vascular damage likely to affect perfusion, we used WMH volume as a covariate in all analyses.

### 2.9 Aortic exam

As previously described [44,46], during the MRI session a thoracic aortic exam was also acquired using simultaneous brachial pressure recording (Model 53,000, Welch Allyn, Skaneateles Falls, NY USA) using a 24- element spine matrix coil. Black blood turbo spin echo sagittal oblique images were acquired to visualize the aortic arch (TR/TE/flip angle: 700 ms/6.5 ms/180°, 1.4 x1.4 mm^2^ in-plane resolution, 2 slices at 7.0 mm). A perpendicular plane to the ascending and descending aorta was defined from these images. A cine phase-contrast velocity encoded series was collected (TR/TE/flip angle: 28.6 ms/1.99 ms/30°, 1.5x1.5x5.5 mm^3^) during 60 cardiac cycles in three segments, with velocity encoding of 180 cm/s through plane. A series of cine FLASH images were acquired within the same plane with the following parameters: TR/TE/flip angle: 59 ms/3.44 ms/15°, with 1.2 x1.2 mm^2^ in-plane resolution and a single slice of 6 mm, 60 cardiac phases, acquired in 8 segments. The aortic data was analyzed using the ARTFUN software [55], where pulse wave velocity in the aortic arch was computed between the ascending and descending aorta from cine phase contrast images. The aortic lumen contours of the ascending and descending aorta were automatically segmented using amplitude images of cine phase contrast series where flow profiles were also estimated. PWV was calculated as described in [44,46].

### 2.10 Statistical analysis

Statistical analysis of all data was done using IBM SPSS Statistics for Windows, Version 24.0 (IBM Corp., Armonk, NY). Descriptive statistics for age, education, MMSE scores, WMH, CBF-CVR, PWV and executive functioning scores are reported in the whole sample and compared between males and females in Table 1. Statistical comparisons between males and females were done using independent samples *t*-tests. Moderation analyses were performed using the PROCESS Macro for moderation analyses [56]. The analyses were bootstrapped to consider any shortcoming in power by simulating greater data based on an algorithm to maintain the current pattern. By default, bootstrapped samples were set to simulate 5,000 samples [56]. Moderation effects of sex on the PWV-CVR, PWV-EF, and CVR-EF relations were tested controlling for age and WMH volume.

**Table 1:** Participant demographics.

Demographic	All (n = 48)	Males (n = 17)	Females (n = 31)
**Age (years)**	63.55 ± 4.86	64 ± 4.37	63 ± 5.14
**Education (years)**	16.29 ± 3.56	16.18 ± 3.32	16.3 ± 53.74
EF*(composite score)	0.003 ± 0.74	-0.31 ± 0.87*	0.177 ± 0.60*
**PWV (m/s)**	8.70 ± 2.89	8.89 ± 2.99	8.59 ± 2.88
**MMSE (out of 30)**	28.79 ± 0.94	28.53 ± 1.32	28.94 ± 0.63
**Log WMH volume**	0.35 ± 0.15	0.36 ± 0.13	0.35 ± 0.17
Resting CBF (ml/100g/min) *	42.46 ± 10.05	35.74 ± 8.71*	46.14 ± 8.85*
**CBF-CVR (ml/100g/min/mmHg CO2)**	4.64 ± 2.39	4.50 ± 2.52	4.71 ± 2.36
**Total intracranial volume (cm3)**	1461.75 ± 119.00	1545.79 ± 116.81	1415.68 ± 93.34

*p<0.05.

### 2.11 Data and code availability

Raw data can be shared as part of a collaboration, as allowed by the informed consent used in this study. The preprocessing scripts along with scripts used to analyze data can be made available upon reasonable request.

## 3. Results

A total of 48 older adults (31 females, 17 males) were included in the analysis. Participant characteristics are summarized in Table 1. It was found that females had a significantly higher resting CBF (p = 0.0001) in whole brain grey matter, and higher composite scores for executive functioning (p = 0.026). There was no difference between males and females for PWV (p > 0.05) and CBF-CVR (p > 0.05) in whole brain grey matter. In addition, adding outliers to this analysis yielded equivalent results for all analyses.

### 3.1 Moderation analysis

**Fig 1:**
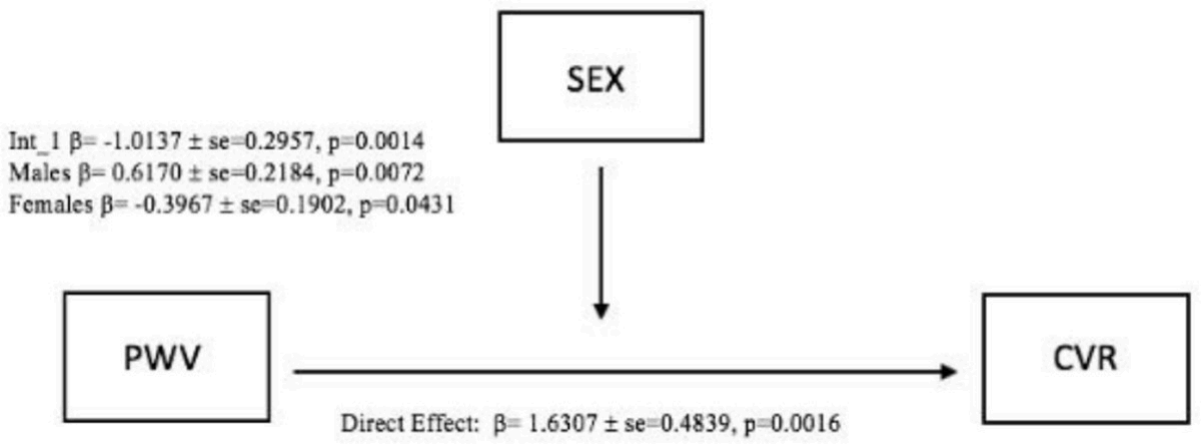
Moderation effect (PWV*SEX) on CVR.

**Fig 2:**
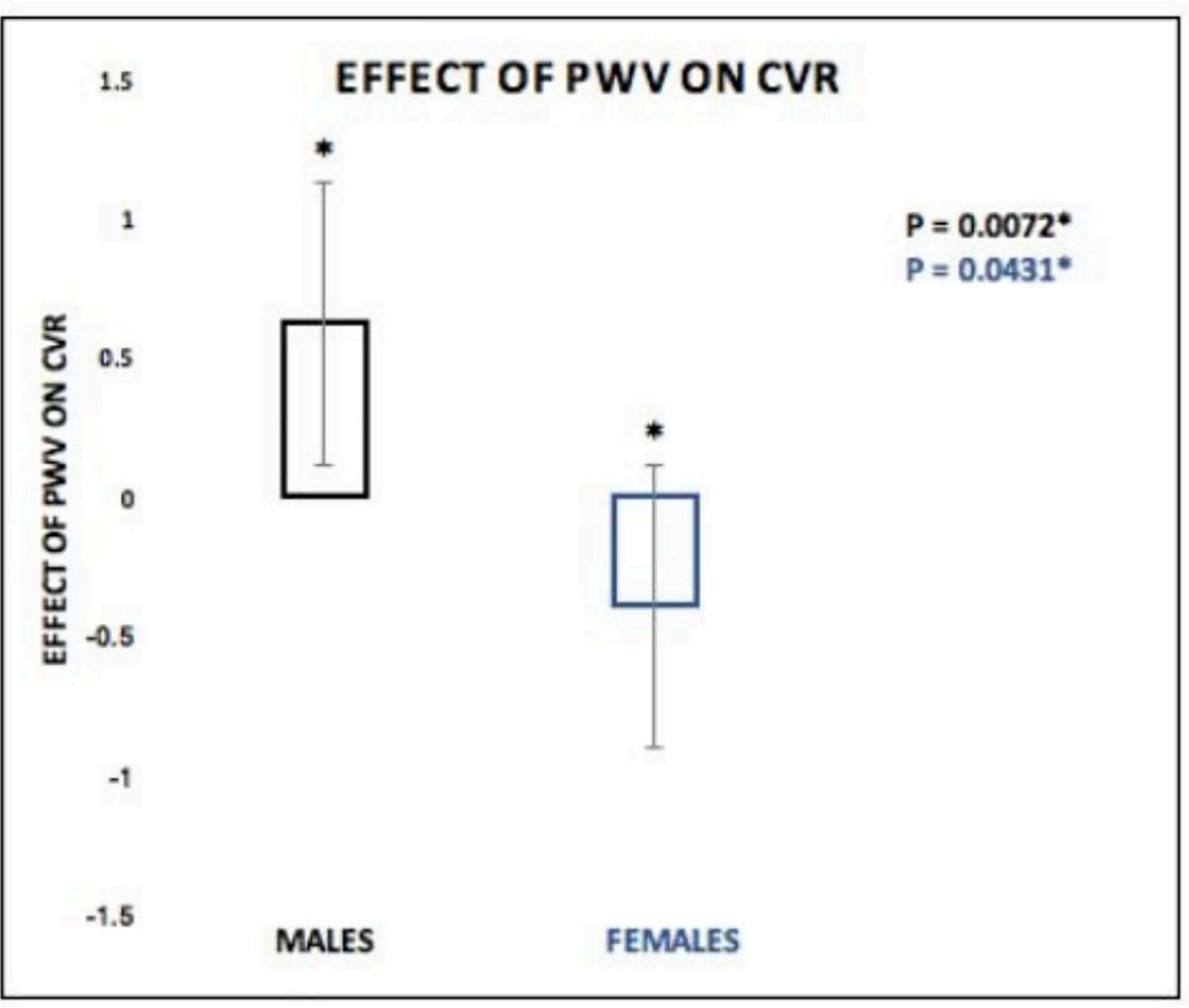
Effect of PWV on CVR. CVR was significantly positive in males (p = 0.0072*) and significantly negative in females (p = 0.0431).

Our results revealed a significant standardized direct effect of PWV on CVR (β = 1.6307, SE = 0.4839, 95% CI [0.654, 2.607], p = 0.0016) as depicted in Fig 1. The moderation effect (SEX *PWV) was also a significant predictor of CVR (β = -1.013, SE = 0.2957, 95% CI [-1.610, -0.4169], p = 0.0014) showing that the effect of PWV on CVR was a function of sex. Further analysis revealed that the effect of PWV on CVR was significantly positive in males (β = 0.6170, SE = 0.2184,95% CI [0.1762, 1.0577], p = 0.0072) and significantly negative in females (β = -0.3967, SE = 0.1902, 95% CI [-0.7805, -0.0129], p = 0.0431) (Fig 2).

**Fig 3:**
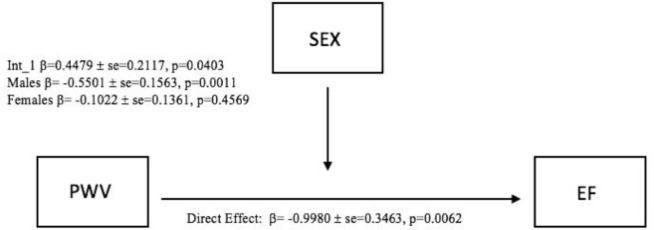
Moderation effect (PWV*SEX) on EF.

**Fig 4:**
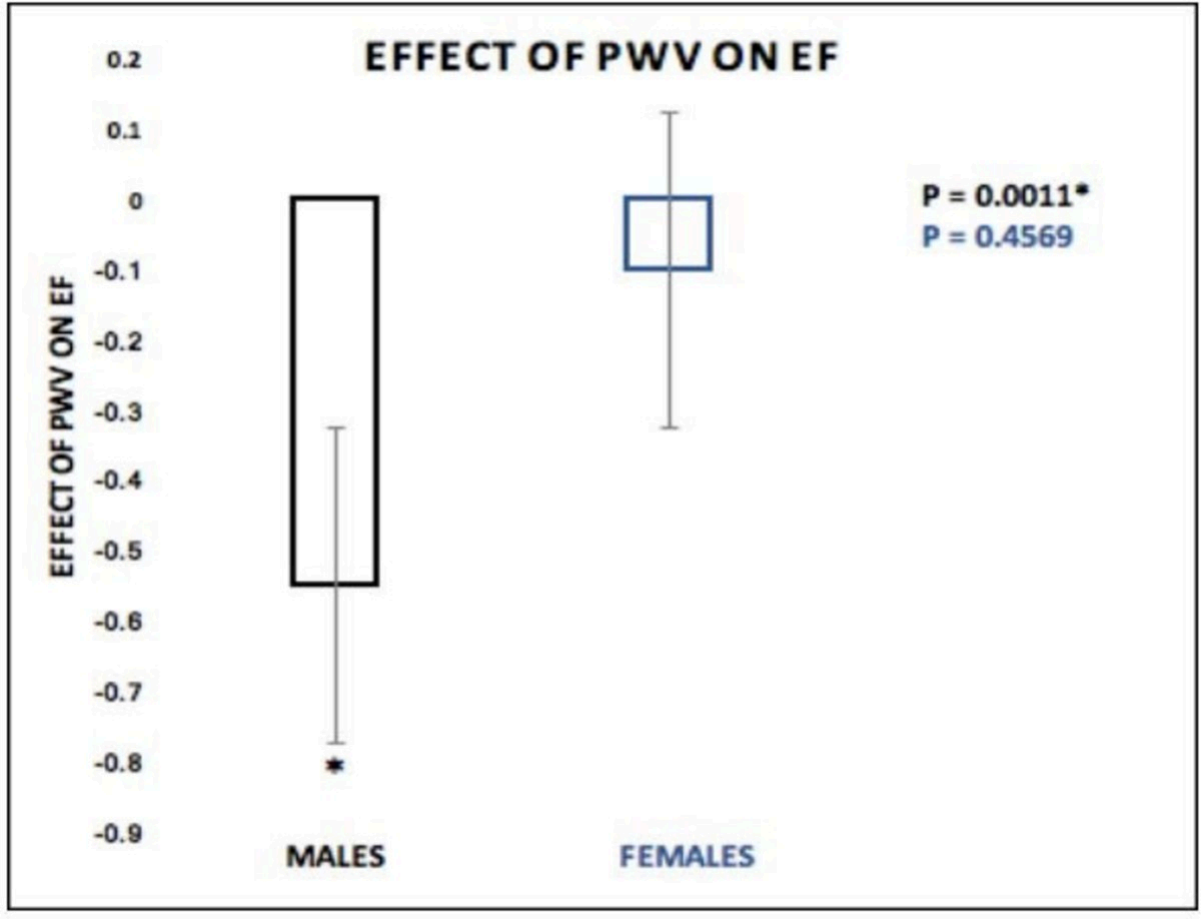
Effect of PWV on EF: The effect of PWV on EF was significantly negative in males (B = -0.5501, SE = 0.1563, 95% CI [-0.8656, -0.2346], p = 0.0011) but not significant in females (B = -0.1022, SE = 0.1361, 95% CI [-0.3769, 0.1725], p = 0.4569).

Our results also revealed a significant standardized direct effect of PWV on EF (β = -0.9980, SE = 0.3463, 95% CI [-1.6970, -0.2990], p = 0.0062). The moderation effect (SEX *PWV) was also a significant predictor of EF (β = 0.4479, SE = 0.2117, 95% CI [0.0207, 0.8751], p = 0.0403) showing that the effect of PWV on EF was a function of sex (Fig 3). As shown in Fig 4 the effect of PWV on EF was significantly negative in males (β = -0.5501, SE = 0.1563, 95% CI [-0.8656, -0.2346], p = 0.0011) but not significant in females (β = -0.1022, SE = 0.1361, 95% CI [-0.3769, 0.1725], p = 0.4569).

**Fig 5:**
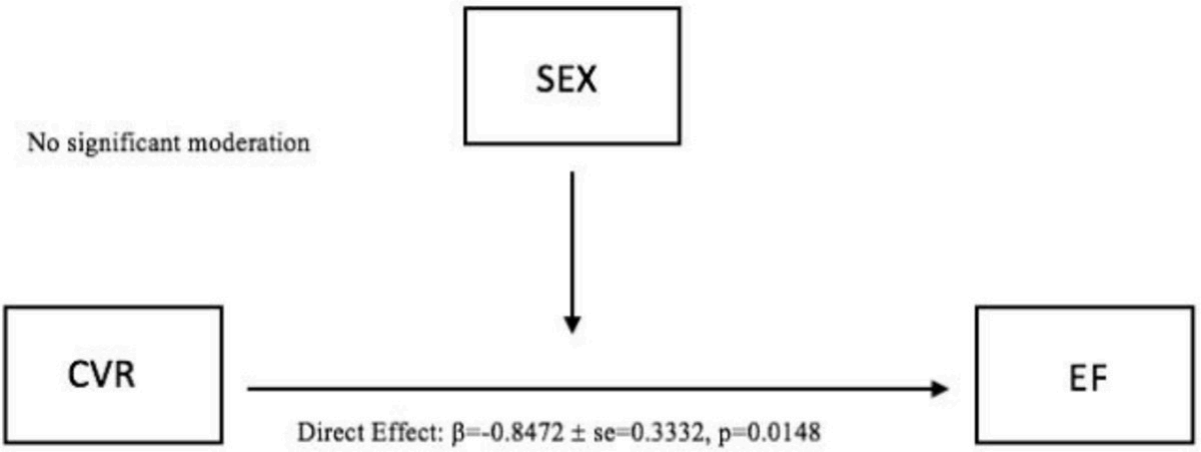
Moderation model (CVR*SEX) on EF.

Finally, a moderation analysis also revealed a significant standardized direct effect of CVR on EF (β = -0.8472, SE = 0.3332, 95% CI [-1.5195, -0.1748], p = 0.0148). However, the moderation effect (SEX *CVR) did not predict EF (β = 0.3438, SE = 0.1990, 95% CI [-0.0579, 0.7455], p = 0.0914) (Fig 5).

## 4. Discussion

### 4.1 Main results

In this study, we investigated the impact of sex on the link between PWV, cognitive performance and CVR. An important finding is that sex moderates the relationship between i) PWV and CVR; and ii) PWV and EF; but not between iii) CVR and EF. Specifically, the effect of PWV on CVR was significantly positive in males and significantly negative in females. Additionally, the effect of PWV on EF was significantly negative in males but not significant in females. Together, our results indicate that some of the complex relationships between PWV, CVR and EF shown in the literature are being driven by sex

### 4.2 Sex differences

The sex differences identified in this study are consistent with the existing literature, including differences in CBF and EF performance. In this sample, females have higher resting CBF than males. These findings are similar to previous studies in healthy older adults indicating that females typically display greater resting global CBF [57,58] and higher CBF velocities compared to males [59–62]. In addition, several studies have reported data suggesting that males and females tend to present with different levels of performance in certain cognitive domains [42,63,64]. Indeed, our findings of lower executive function scores in older males compared to older females are also in line with previous research [41].

### 4.3 Arterial stiffness and cerebrovascular reactivity

Overall, there is substantial evidence supporting an effect of sex hormones on vascular stiffness [5,65]. Over the lifespan, arterial stiffness increases linearly in both males and females however there is a more rapid increase in stiffness in females due to depletions in estrogen levels post menopause [5,66]. Indeed, several studies have shown that hormone receptors, including estrogen and testosterone are cardio-protective [5,67–69]. Previous work highlighting the protective effects of estrogen have shown that arterial stiffness, measured using cfPWV, is reduced in postmenopausal females taking hormone replacement therapy (HRT) when compared to matched females not taking HRT [5,70]. In addition, testosterone was once thought to play a role in promoting CVD among males [71,72], however recent epidemiological studies point to the contrary. Testosterone deficiency has been associated with increased cfPWV among healthy older males compared to age-matched males with normal testosterone [5,73]. In addition, low testosterone has also been associated with impaired microvascular function and arterial elasticity, measured using the augmentation index [5,12]. Finally, it is known that testosterone can be converted to estrogen in the brain, so that the protective effects of estrogen may in fact be better preserved in older males than females [74]. Overall, ample data suggest that sex hormones substantially impact the manifestation of arterial stiffness among males and females. Given the age of our sample (average 63 years) and the average age of naturally occurring menopause (51 years), it is likely that most of our female sample was post-menopausal, and therefore expressing very little sex hormones and sex hormone receptors.

Although quantitative measurements of CBF and CVR in relation with arterial stiffness have been performed by others [30,31], the literature shows conflicting results. While more recent reports suggest a preserved CVR with increased aortic stiffness [31,33], earlier studies using PET and TCD rather than MRI did not find such an association, but rather reductions in CVR among adults with greater aortic stiffness [30,32]. It is possible however that these counterintuitive findings could stem from differences in study designs, heterogeneity of studied populations (males vs females) and/or differences in imaging modalities. For example, Dubose et al (2018) [30] and Jaruchart et al. (2016) [32] both had a population of 40 older adults with only 11 females whereas our study had more females than males. In addition, one previous TCD study describing sex-related differences in cerebral vasomotor reactivity has shown increased vasodilatory response in females compared with male subjects [75]. More importantly, these studies did not control for menstrual phase or account for sex hormones, which are known to acutely alter CBF and cerebrovascular responsiveness [76–80] Our work presents the first evidence of a sex moderation in the relationship between PWV and CVR. Our findings of low CVR among females and high CVR among males with increased arterial stiffness are in line with the contradictory results found in the extant literature, with females following the trend observed by DuBose et al and Jaruchart et al. [30,32] and male data being in agreement with the results of Jefferson et al. [31]. The biological underpinnings of these differences may be of several origins and yet there is little data to support a particular hypothesis. However, it could be differential remodeling or damage as a function of stiffness may depend on the time course of stiffness (with men having a potentially more protracted time course since they do not undergo menopause) or may be due to different biases in the CVR technique in males and females. ASL is highly dependent on transit time of the tag for example, and differences in velocity and transit time could for example explain some of these effects. More studies investigating potential sex-dependent methodological biases, and the biological changes that underpin vascular changes in aging in both sexes are needed to start addressing this question. Following these and our results, it is imperative that different models should be created for sex rather than just regressing out the effects of sex.

### 4.4 The association between pulse wave velocity and cognitive function relative to sex

In the present study, the relationship between arterial stiffness and EF was in part driven by differences in sex. We also demonstrate that higher arterial stiffness, as measured by PWV, is associated with poorer performance on EF tasks among males only. Our findings are in line with prior studies that have demonstrated sex differences in the relationship between arterial stiffness and cognitive function. For instance, Singer et al. found no significant association between arterial stiffness and global cognition, however negative associations between PWV and composite global cognition were observed in males only when stratifying the sample by sex [27]. Similarly, Waldstein et al reported males as scoring significantly lower than females on tests of verbal memory at higher levels of pulse pressure, an alternative measure of arterial stiffness [81]. In addition, there is good agreement in the literature showing that arterial stiffness and related central hemodynamics are associated with reductions in cognitive performance on memory, processing speed, and executive function tasks [15,17,34]. However, these studies are limited because they have used a nonspecific measure of cognition (i.e., MMSE). Here, we extend their findings to include executive function domains that are especially susceptible to cardiovascular disease [82]. Indeed, our results are consistent with other studies pointing to a general association between PWV and executive dysfunction [37,83,84]. For example, Hajjar et al found that subjects with higher PWV had the greatest 4-year risk of decline in executive function [84]. It has been hypothesized that this may be due to the fact that the dorsolateral prefrontal cortex, essential for executive function tasks, is situated in a watershed region of the brain [85,86]. Thus, one could speculate that with increased arterial stiffness, these regions may be deprived of perfusion and therefore oxygen earlier than better perfused regions [85,86].

Our results showed no significant association between arterial stiffness and EF among females. Moreover, females in this cohort displayed significantly better performance than males on the cognitive tasks assessed. It is well known that there are significant differences between sexes regarding vascular function and cognitive performance among older adults [42,87]. Indeed, across the lifespan, males have a higher risk for CVDs and females are relatively protected until menopause after which they slowly catch up to males over about two decades [27,87]. A possible hypothesis for the sex differences observed here is therefore that males experience declines earlier in life, while the postmenopausal-related cognitive changes are still very mild among our population of women, who are on average within 12-year of the average menopause age within the general population and may take more time to mimic the changes observed in our males.

### 4.5 The association between cognitive function and cerebrovascular reactivity relative to sex

While the vessel reactivity-cognition link has been investigated in cardiovascular and neurodegenerative disorders, the association between CVR and cognition in healthy aging is unclear. Most reports indicate that blood vessel reactivity in the brain decreases with healthy aging [44,45,52,53,88–91]. The mechanisms underlying this decreasing reactivity of cerebral vessels with aging are postulated to be related to local vascular stiffening [92]. An interesting finding of our study is that sex did not moderate the relationship between CVR and EF among older adults. This is perhaps partly attributable to the fact that CBF-CVR and PWV did not significantly differ between our male and female population. Interestingly, recent work published by our group has shown that CVR, which quantifies the hemodynamic response to a vasodilatory stimulus, may also not be uniquely dependent on brain-based vascular properties such as vascular elasticity, and may be partly dependent on more global properties such as changes in endothelial function, chemosensitivity and cerebral autoregulation [46]. As such, CVR should be interpreted with caution as it is likely to reflect various physiological parameters, including stiffness, endothelial health, chemosensitivity, and perhaps more global components reflecting the role of the pressure wave in vasodilation. Nonetheless, future work aiming to disentangle the relationships between CVR, and cognition should implement more robust and direct measures of vascular elasticity in the brain [93,94].

### 4.6 Limitations

The results from this study should be viewed considering some limitations. First, menopausal status and hormonal levels were not acquired at the time of the study. It is however reasonable to assume that our female population is predominantly postmenopausal given that the age range was 55–75 years, with a mean age of 63. In fact, the average age of naturally occurring menopause in Canada is 51 ±4.4 years (Canadian consensus conference on menopause, 2006). In this context, it is likely that sex hormone levels were low in our female sample. Nonetheless, this assumption is speculative, and other studies should account for menopausal status to replicate and provide clarity on the mechanisms that underlie the present results. Future work should include younger subjects (40–55 years of age) to better study hormonal status in females.

While a strength of the current study is the use of non-invasive quantitative perfusion imaging to calculate cerebral blood flow and CVR, one must note that the ASL technique suffers from several limitations. The contrast afforded by the subtraction of tagged images is only a fraction of a percent of the functional MRI contrast, providing limited signal-to-noise ratio (SNR) [17,95]. Furthermore, full coverage of the brain is typically not possible without advanced multi-band approaches, limiting the ability to draw conclusions on the entire brain [17,95]. Finally, most ASL imaging methods are unable to determine whether the changes detected are true reflections of changes in flow or the result of alterations in transit times [17]. This is especially problematic in the case of single delay ASL studies, such as the one presented here. Despite these limitations of ASL, it has consistently been shown that CBF-CVR is a more specific measure of vascular health than BOLD-CVR [96]. Indeed, CBF-CVR is more sensitive to vascular elasticity than BOLD, and may be the best choice when sensitivity is desired over specificity [96]. In addition to the general limitations of ASL, the post-labeling delay chosen in this study is suboptimal for older adults since it was optimized for a younger population [17,44,46,97]. As such, with our limited SNR, it is possible that our CBF data before and during hypercapnia was underestimated. Thus, to better understand the relationship between arterial stiffness, cognition and CVR, longitudinal studies using multi-band approaches and multi-delay implementations with optimized post-labeling delays are necessary.

Another limitation of our study is the lack of comparison with healthy younger adults. Although data was collected among a younger population, when quantifying the standard deviation for pulse wave velocity and cognition, younger adults had lower variance compared to older adults for PWV and cognition. Because of this lower variance, we did not expect that any of these relationships or sex differences would exist among younger adults. An initial moderation test confirmed that these sex differences were not present at a younger age. Future studies could however explore these effects in a larger sample with a wider range of health status to assess whether similar effects could be observed in a younger population.

A further limitation to this study is our sample size. Indeed, our subset of female and male participants is small, limiting our ability to fully generalize our findings to the general population. Furthermore, it may be plausible that due to our small sample size we were unable to detect the mediating effect of sex in some of our models. In addition, our ratio of males (n = 17) to females (n = 31) in this sample may be biased toward females, underrepresenting our male population. As such, our study may have limited statistical power to detect an effect size of practical importance, especially in males. Nonetheless, our analyses were bootstrapped which can overcome the power problem of small samples. Also, our sample included predominantly very healthy older adults, limiting our ability to generalize our findings to the general population of older adults, who often suffer from cardiovascular risk factors. We speculate that associations reported here would likely be stronger in a cohort with worse cardiovascular health. Moreover, the present findings should be confirmed in larger populations. Our analyses can be considered as exploratory results given the small number of participants involved and the fact that our participants were healthy as compared to large segments of the general population. However, even if generalization should be confirmed by further research, we believe that the present findings should alert professionals in the field of cardiovascular and neurological health about the high probability of sex differences in the relationship between PWV and EF and PWV and CVR.

Finally, this study has a cross sectional design making it difficult to draw general conclusions about the effects of stiffening in isolation of other possible co-occurring factors. Although this study provides valuable knowledge on the impact of sex on the relationship between aortic stiffness, cognition and CVR, a longitudinal study is needed to better understand the sex differences among those hemodynamic measures across the lifespan to better personalize CVD prevention strategies.

## 5. Conclusion

The findings from this study add to a growing body of research that underscores the interrelationship between cardiovascular function and brain health among aging adults. Overall, this paper identified a sex moderation between PWV and CVR, PWV and EF but not between CVR and EF in a sample of healthy older adults. These findings could be the results of different sex hormones, such as estrogen and testosterone, that are known to alter cerebrovascular measures of brain health. More importantly, understanding these hemodynamic associations may lead to earlier detection and targeted interventions to prevent or lessen the onset of cardiovascular diseases linked with higher aortic stiffening. Thus, future longitudinal studies that explore sex differences should include evaluation of the role of hormone variations (i.e., sex hormones).

## Supporting information

S1 Fig Values reported as Z scores showing a positive relationship between cerebrovascular reactivity (CVR) and pulse wave velocity (PWV) among men (r = 0.551; p = 0.027). (TIF)

S2 Fig Values reported as Z scores showing a negative relationship between pulse wave velocity (PWV) and executive function among men (r = -0.659; p = 0.006). (TIF)

S3 Fig Values reported as Z scores showing a negative relationship between executive function and cerebrovascular reactivity (CVR) among men (r = -0.640; p = 0.008). (TIF)

## References

[1] Members W. G., Lloyd-Jones D., Adams R. J., Brown T. M., Carnethon M., Dai S., et al. (2010). Executive summary: heart disease and stroke statistics—2010 update: a report from the American Heart Association. *Circulation*, 121(7), 948–954. doi: 10.1161/CIRCULATIONAHA.109.192666 20177011

[2] Appelros P., Stegmayr B., & Terént A. (2009). Sex differences in stroke epidemiology: a systematic review. Stroke; a Journal of Cerebral Circulation, 40(4), 1082–1090. 10.1161/STROKEAHA.108.540781 19211488

[3] Coutinho T. (2014). Arterial stiffness and its clinical implications in women. The Canadian Journal of Cardiology, 30(7), 756–764. doi: 10.1016/j.cjca.2014.03.020 24970788

[4] Ellekjaer H., Holmen J., Indredavik B., & Terent A. (1997). Epidemiology of stroke in Innherred, Norway, 1994 to 1996. Incidence and 30-day case-fatality rate. Stroke; a Journal of Cerebral Circulation, 28(11), 2180–2184. 10.1161/01.str.28.11.2180 9368561

[5] DuPont J. J., Kenney R. M., Patel A. R., & Jaffe I. Z. (2019a). Sex differences in mechanisms of arterial stiffness. British Journal of Pharmacology. 10.1111/bph.14624 PMC6877796 30767200

[6] Maas A. H. E. M., A H E, & Appelman Y. E. A. (2010a). Gender differences in coronary heart disease. Netherlands Heart Journal, Vol. 18, pp. 598–603. 10.1007/s12471-010-0841-y 21301622 PMC3018605

[7] Towfighi A., Zheng L., & Ovbiagele B. (2009). Sex-specific trends in midlife coronary heart disease risk and prevalence. Archives of Internal Medicine, 169(19), 1762–1766. doi: 10.1001/archinternmed.2009.318 19858433

[8] Pepine C. J., Nichols W. W., & Pauly D. F. (2006). Estrogen and different aspects of vascular disease in women and men. Circulation Research, 99(5), 459–461. doi: 10.1161/01.RES.0000241056.84659.59 16946140

[9] Orshal J. M., & Khalil R. A. (2004). Gender, sex hormones, and vascular tone. American Journal of Physiology. Regulatory, Integrative and Comparative Physiology, 286(2), R233–R249 doi: 10.1152/ajpregu.00338.2003 14707008

[10] Shaw L. J., Merz C. N. B., Pepine C. J., Reis S. E., Bittner V., Kelsey S. F., et al. (2006). Insights from the NHLBI-Sponsored Women’s Ischemia Syndrome Evaluation (WISE) Study: Part I: gender differences in traditional and novel risk factors, symptom evaluation, and gender-optimized diagnostic strategies. Journal of the American College of Cardiology, 47(3 Supplement), S4–S20. 16458170 10.1016/j.jacc.2005.01.072

[11] Kyriazis J., Tzanakis I., Stylianou K., Katsipi I., Moisiadis D., Papadaki A., et al. (2011). Low serum testosterone, arterial stiffness, and mortality in male haemodialysis patients. Nephrology, Dialysis, Transplantation: Official Publication of the European Dialysis and Transplant Association—European Renal Association, 26(9), 2971–2977. 10.1093/ndt/gfq847 21427069

[12] Corrigan F. E. 3rd, Al Mheid I., Eapen D. J., Hayek S. S., Sher S., Martin G. S., et al. (2015). Low testosterone in men predicts impaired arterial elasticity and microvascular function. International Journal of Cardiology, 194, 94–99. doi: 10.1016/j.ijcard.2015.05.065 26022684 PMC9135451

[13] Mitchell G. F. (2009). Arterial Stiffness and Wave Reflection: Biomarkers of Cardiovascular Risk. Artery Research, 3(2), 56–64. doi: 10.1016/j.artres.2009.02.002 20161241 PMC2705910

[14] Scuteri A., Brancati A. M., Gianni W., Assisi A., & Volpe M. (2005). Arterial stiffness is an independent risk factor for cognitive impairment in the elderly: a pilot study. Journal of Hypertension, 23(6), 1211–1216. doi: 10.1097/01.hjh.0000170384.38708.b7 15894897

[15] Iulita M. F., Noriega de la Colina A., & Girouard H. (2018). Arterial stiffness, cognitive impairment, and dementia: confounding factor or real risk? Journal of Neurochemistry, 144(5), 527–548. doi: 10.1111/jnc.14235 28991365

[16] Badji A., de la Colina A. N., Sabra D., Karakuzu A., Bherer L., Lamarre-cliche M., et al. (2019). The relationship between cognitive function, cortical blood flow and sub-cortical white-matter health in the elderly. JOURNAL OF CEREBRAL BLOOD FLOW AND METABOLISM, 39, 29–30. SAGE PUBLICATIONS INC 2455 TELLER RD, THOUSAND OAKS, CA 91320 USA.

[17] Badji A., Sabra D., Bherer L., Cohen-Adad J., Girouard H., & Gauthier C. J. (2019). Arterial Stiffness and Brain Integrity: A review of MRI findings. Ageing Research Reviews. doi: 10.1016/j.arr.2019.05.001 31063866

[18] Novak V. (2012a). Cognition and Hemodynamics. Current Cardiovascular Risk Reports, 6(5), 380–396. doi: 10.1007/s12170-012-0260-2 23050027 PMC3462450

[19] Van Bortel L. M., Laurent S., Boutouyrie P., Chowienczyk P., Cruickshank J. K., De Backer T., et al (2012). Expert consensus document on the measurement of aortic stiffness in daily practice using carotid-femoral pulse wave velocity. Journal of hypertension, 30(3), 445–448. doi: 10.1097/HJH.0b013e32834fa8b0 22278144

[20] Rossi P., Francès Y., Kingwell B. A., & Ahimastos A. A. (2011). Gender differences in artery wall biomechanical properties throughout life. Journal of Hypertension, 29(6), 1023–1033. doi: 10.1097/HJH.0b013e328344da5e 21346620

[21] Segers P., Rietzschel E. R., De Buyzere M. L., Vermeersch S. J., De Bacquer D., Van Bortel L. M., et al. (2007a). Noninvasive (Input) Impedance, Pulse Wave Velocity, and Wave Reflection in Healthy Middle-Aged Men and Women. Hypertension, Vol. 49, pp. 1248–1255. 10.1161/hypertensionaha.106.085480. 17404183

[22] Qiu H., Depre C., Ghosh K., Resuello R. G., Natividad F. F., Rossi F., et al. (2007). Mechanism of gender-specific differences in aortic stiffness with aging in nonhuman primates. Circulation, 116(6), 669–676. doi: 10.1161/CIRCULATIONAHA.107.689208 17664374

[23] Kim J.-Y., Park J. B., Kim D. S., Kim K. S., Jeong J. W., Park J. C., et al. (2014). Gender Difference in Arterial Stiffness in a Multicenter Cross-Sectional Study: The Korean Arterial Aging Study (KAAS). The Pulse of the Montana State Nurses’ Association, 2(1–4), 11–17. 10.1159/000365267 PMC4646149 26587439

[24] Regnault V., Thomas F., Safar M. E., Osborne-Pellegrin M., Khalil R. A., Pannier B., et al. (2012). Sex difference in cardiovascular risk: role of pulse pressure amplification. Journal of the American College of Cardiology, 59(20), 1771–1777. doi: 10.1016/j.jacc.2012.01.044 22575315 PMC3716253

[25] Pase M. P., Himali J. J., Mitchell G. F., Beiser A., Maillard P., Tsao C., et al. (2016). Association of Aortic Stiffness with Cognition and Brain Aging in Young and Middle-Aged Adults: The Framingham Third Generation Cohort Study. Hypertension, 67(3), 513–519. doi: 10.1161/HYPERTENSIONAHA.115.06610 26754644 PMC4752398

[26] O’Rourke M. F., & Safar M. E. (2005). Relationship between aortic stiffening and microvascular disease in brain and kidney: cause and logic of therapy. Hypertension, 46(1), 200–204. doi: 10.1161/01.HYP.0000168052.00426.65 15911742

[27] Singer J., Trollor J. N., Crawford J., O’Rourke M. F., Baune B. T., Brodaty H., et al. (2013a). The association between pulse wave velocity and cognitive function: the Sydney Memory and Ageing Study. PloS One, 8(4), e61855. doi: 10.1371/journal.pone.0061855 23637918 PMC3640074

[28] TarumiT., GonzalesM. M., FallowB., NualnimN., PyronM., TanakaH., et al. (2013b). Central artery stiffness, neuropsychological function, and cerebral perfusion in sedentary and endurance-trained middle-aged adults. Journal of Hypertension, 31(12), 2400–2409. doi: 10.1097/HJH.0b013e328364decc 24220591

[29] Tarumi T., Shah F., Tanaka H., & Haley A. P. (2011). Association between central elastic artery stiffness and cerebral perfusion in deep subcortical gray and white matter. American Journal of Hypertension, 24(10), 1108–1113. doi: 10.1038/ajh.2011.101 21654859

[30] DuBose L. E., Boles Ponto L. L., Moser D. J., Harlynn E., Reierson L., & Pierce G. L. (2018). Higher Aortic Stiffness Is Associated with Lower Global Cerebrovascular Reserve Among Older Humans. Hypertension, 72(2), 476–482. doi: 10.1161/HYPERTENSIONAHA.118.11143 29915015 PMC6261448

[31] Jefferson A. L., Cambronero F. E., Liu D., Moore E. E., Neal J. E., Terry J. G., et al. (2018). Higher Aortic Stiffness is Related to Lower Cerebral Blood Flow and Preserved Cerebrovascular Reactivity in Older Adults. Circulation. doi: 10.1161/CIRCULATIONAHA.118.032410 30018169 PMC6394409

[32] Jaruchart T., Suwanwela N. C., Tanaka H., & Suksom D. (2016). Arterial stiffness is associated with age-related differences in cerebrovascular conductance. Experimental Gerontology, Vol. 73, pp. 59–64. doi: 10.1016/j.exger.2015.11.006 26571202

[33] Zhu Y.-S., Tarumi T., Tseng B. Y., Palmer D. M., Levine B. D., & Zhang R. (2013). Cerebral vasomotor reactivity during hypo- and hypercapnia in sedentary elderly and master’s athletes. Journal of Cerebral Blood Flow and Metabolism: Official Journal of the International Society of Cerebral Blood Flow and Metabolism, 33(8), 1190–1196. 10.1038/jcbfm.2013.66 PMC3734768 23591649

[34] Singer J., Trollor J. N., Baune B. T., Sachdev P. S., & Smith E. (2014). Arterial stiffness, the brain and cognition: a systematic review. Ageing Research Reviews, 15, 16–27. doi: 10.1016/j.arr.2014.02.002 24548924

[35] Elias M. F., Robbins M. A., Budge M. M., Abhayaratna W. P., Dore G. A., & Elias P. K. (2009a). Arterial pulse wave velocity and cognition with advancing age. Hypertension, 53(4), 668–673. 19237680 10.1161/HYPERTENSIONAHA.108.126342 PMC2716128

[36] Fukuhara M., Matsumura K., Ansai T., Takata Y., Sonoki K., Akifusa S., et al. (2006a). Prediction of cognitive function by arterial stiffness in the very elderly. Circulation Journal: Official Journal of the Japanese Circulation Society, 70(6), 756–761. doi: 10.1253/circj.70.756 16723799

[37] Poels M. M. F., van Oijen M., Mattace-Raso F. U. S., Hofman A., Koudstaal P. J., Witteman J. C. M., et al. (2007b). Arterial stiffness, cognitive decline, and risk of dementia: the Rotterdam study. Stroke; a Journal of Cerebral Circulation, 38(3), 888–892. doi: 10.1161/01.STR.0000257998.33768.87 17272780

[38] Watson N. L., Sutton-Tyrrell K., Rosano C., Boudreau R. M., Hardy S. E., Simonsick E. M., et al. (2011a). Arterial stiffness and cognitive decline in well-functioning older adults. The Journals of Gerontology. Series A, Biological Sciences and Medical Sciences, 66(12), 1336–1342. doi: 10.1093/gerona/glr119 21768503 PMC3210954

[39] Silvestrini M., Pasqualetti P., Baruffaldi R., Bartolini M., Handouk Y., Matteis M., et al. (2006a). Cerebrovascular reactivity and cognitive decline in patients with Alzheimer disease. Stroke; a Journal of Cerebral Circulation, 37(4), 1010–1015. doi: 10.1161/01.STR.0000206439.62025.97 16497984

[40] Haratz S., Weinstein G., & Molshazki N. (2015). Impaired cerebral hemodynamics and cognitive performance in patients with atherothrombotic disease. Journal of. Retrieved from doi: 10.3233/JAD-150052 25720410 PMC5753416

[41] Halpern D. F., & LaMay M. L. (2000). The Smarter Sex: A Critical Review of Sex Differences in Intelligence. Educational Psychology Review, 12(2), 229–246.

[42] Castonguay N., Lussier M., Bugaiska A., Lord C., & Bherer L. (2015). Executive functions in men and postmenopausal women. Journal of Clinical and Experimental Neuropsychology, 37(2), 193–208. doi: 10.1080/13803395.2014.1000267 25695230

[43] Kurlowicz L., & Wallace M. (1999). The Mini-Mental State Examination (MMSE). Journal of Gerontological Nursing, 25(5), 8–9. doi: 10.3928/0098-9134-19990501-08 10578759

[44] Gauthier C. J., Lefort M., Mekary S., Desjardins-Crépeau L., Skimminge A., Iversen P., et al. (2015a). Hearts and minds: linking vascular rigidity and aerobic fitness with cognitive aging. Neurobiology of Aging, 36(1), 304–314. 25308963 10.1016/j.neurobiolaging.2014.08.018

[45] Gauthier C. J., Madjar C., Desjardins-Crépeau L., Bellec P., Bherer L., & Hoge R. D. (2013a). Age dependence of hemodynamic response characteristics in human functional magnetic resonance imaging. Neurobiology of Aging, 34(5), 1469–1485. 23218565 10.1016/j.neurobiolaging.2012.11.002

[46] Intzandt B., Sabra D., Foster C., Desjardins-Crépeau L., Hoge R. D., Steele C. J., et al. (2019). Higher cardiovascular fitness level is associated with lower cerebrovascular reactivity and perfusion in healthy older adults. Journal of Cerebral Blood Flow & Metabolism, p. 0271678X1986287. doi: 10.1177/0271678X19862873 31342831 PMC7308519

[47] Penny W. D., Friston K. J., Ashburner J. T., Kiebel S. J., & Nichols T. E. (2011). Statistical Parametric Mapping: The Analysis of Functional Brain Images. Elsevier.

[48] Ashburner J., & Friston K. J. (2000). Voxel-Based Morphometry—The Methods. NeuroImage, 11(6), 805–821. 10860804 10.1006/nimg.2000.0582

[49] Gaser C. (2016). Structural MRI: Morphometry. In Reuter M. & Montag C. (Eds.), Neuroeconomics (pp. 399–409). Berlin, Heidelberg: Springer Berlin Heidelberg.

[50] Avants B. B., Epstein C. L., Grossman M., & Gee J. C. (2008). Symmetric diffeomorphic image registration with cross-correlation: evaluating automated labeling of elderly and neurodegenerative brain. Medical Image Analysis, 12(1), 26–41. doi: 10.1016/j.media.2007.06.004 17659998 PMC2276735

[51] Liu T. T., & Wong E. C. (2005). A signal processing model for arterial spin labeling functional MRI. NeuroImage, 24(1), 207–215. doi: 10.1016/j.neuroimage.2004.09.047 15588612

[52] Gauthier C. J., & Hoge R. D. (2012a). Magnetic resonance imaging of resting OEF and CMRO2 using a generalized calibration model for hypercapnia and hyperoxia. NeuroImage, 60(2), 1212–1225. 22227047 10.1016/j.neuroimage.2011.12.056

[53] Gauthier C. J., Desjardins-Crépeau L., Madjar C., Bherer L., & Hoge R. D. (2012a). Absolute quantification of resting oxygen metabolism and metabolic reactivity during functional activation using QUO2 MRI. NeuroImage, 63(3), 1353–1363. 22986357 10.1016/j.neuroimage.2012.07.065

[54] Glover G. H. (1999). Deconvolution of Impulse Response in Event-Related BOLD fMRI1. NeuroImage, 9(4), 416–429. doi: 10.1006/nimg.1998.0419 10191170

[55] Herment A., Kachenoura N., Lefort M., Bensalah M., Dogui A., Frouin F., et al. (2010). Automated segmentation of the aorta from phase contrast MR images: validation against expert tracing in healthy volunteers and in patients with a dilated aorta. Journal of Magnetic Resonance Imaging: JMRI, 31(4), 881–888. doi: 10.1002/jmri.22124 20373432

[56] Hayes A. F. (2017). Introduction to Mediation, Moderation, and Conditional Process Analysis, Second Edition: A Regression-Based Approach. Guilford Publications.

[57] Rodriguez G., Warkentin S., Risberg J., & Rosadini G. (1988). Sex differences in regional cerebral blood flow. Journal of Cerebral Blood Flow and Metabolism: Official Journal of the International Society of Cerebral Blood Flow and Metabolism, 8(6), 783–789. doi: 10.1038/jcbfm.1988.133 3192645

[58] Esposito G., Van Horn J. D., Weinberger D. R., & Berman K. F. (1996). Gender differences in cerebral blood flow as a function of cognitive state with PET. Journal of Nuclear Medicine: Official Publication, Society of Nuclear Medicine, 37(4), 559–564. 8691239

[59] Vriens E. M., Kraaier V., Musbach M., Wieneke G. H., & van Huffelen A. C. (1989). Transcranial pulsed doppler measurements of blood velocity in the middle cerebral artery: Reference values at rest and during hyperventilation in healthy volunteers in relation to age and sex. Ultrasound in Medicine & Biology, Vol. 15, pp. 1–8. 10.1016/0301-5629(89)90125-7. 2646803

[60] Martin P. J., Evans D. H., & Naylor A. R. (1994). Transcranial color-coded sonography of the basal cerebral circulation. Reference data from 115 volunteers. Stroke; a Journal of Cerebral Circulation, 25(2), 390–396. doi: 10.1161/01.str.25.2.390 7905680

[61] Oláh L., Valikovics A., Bereczki D., Fülesdi B., Munkácsy C., & Csiba L. (2000). Gender-Related Differences in Acetazolamide-Induced Cerebral Vasodilatory Response: A Transcranial Doppler Study. Journal of Neuroimaging, Vol. 10, pp. 151–156. doi: 10.1111/jon2000103151 10918741

[62] Tegeler C. H., Crutchfield K., Katsnelson M., Kim J., Tang R., Passmore Griffin L., et al. (2013). Transcranial Doppler velocities in a large, healthy population. Journal of Neuroimaging: Official Journal of the American Society of Neuroimaging, 23(3), 466–472. 23157483 10.1111/j.1552-6569.2012.00711.x

[63] Burstein B., Bank L., & Jarvik L. F. (1980). Sex differences in cognitive functioning: evidence, determinants, implications. Human Development, 23(5), 289–313. doi: 10.1159/000272593 7390471

[64] Kennison S. M. (2003). Sex differences in cognitive abilities (3rd edition). Diane F. Halpern. Lawrence Erlbaum Associates, Mahwah, NJ, 2000. No. of pages 420. ISBN 0-8058-2792-7. Price: $39.95. Applied Cognitive Psychology, Vol. 17, pp. 375–376. 10.1002/acp.883.

[65] Ogola B. O., Zimmerman M. A., Clark G. L., Abshire C. M., Gentry K. M., Miller K. S., et al. (2018). New insights into arterial stiffening: does sex matter? American Journal of Physiology. Heart and Circulatory Physiology, 315(5), H1073–H1087. doi: 10.1152/ajpheart.00132.2018 30028199 PMC6415742

[66] Mitchell G. F. (2014). Arterial Stiffness and Hypertension. Hypertension, Vol. 64, pp. 210–214. doi: 10.1161/HYPERTENSIONAHA.114.03449 24799614 PMC4185002

[67] Wu J., Hadoke P. W. F., Mair I., Lim W. G., Miller E., Denvir M. A., et al. (2014). Modulation of neointimal lesion formation by endogenous androgens is independent of vascular androgen receptor. Cardiovascular Research, 103(2), 281–290. doi: 10.1093/cvr/cvu142 24903497 PMC4094672

[68] Karas R. H., Patterson B. L., & Mendelsohn M. E. (1994). Human vascular smooth muscle cells contain functional estrogen receptor. Circulation, 89(5), 1943–1950. doi: 10.1161/01.cir.89.5.1943 8181116

[69] Dockery F., Bulpitt C. J., Donaldson M., Fernandez S., & Rajkumar C. (2003). The relationship between androgens and arterial stiffness in older men. Journal of the American Geriatrics Society, 51(11), 1627–1632. doi: 10.1046/j.1532-5415.2003.51515.x 14687394

[70] Rajkumar C., Kingwell B. A., Cameron J. D., Waddell T., Mehra R., Christophidis N., et al. (1997). Hormonal therapy increases arterial compliance in postmenopausal women. Journal of the American College of Cardiology, 30(2), 350–356. doi: 10.1016/s0735-1097(97)00191-5 9247504

[71] Thompson P. D., Cullinane E. M., Sady S. P., Chenevert C., Saritelli A. L., Sady M. A., et al. (1989). Contrasting effects of testosterone and stanozolol on serum lipoprotein levels. JAMA: The Journal of the American Medical Association, 261(8), 1165–1168. 2915439

[72] Sullivan M. L., Martinez C. M., Gennis P., & John Gallagher E. (1998). The cardiac toxicity of anabolic steroids. Progress in Cardiovascular Diseases, Vol. 41, pp. 1–15. doi: 10.1016/s0033-0620(98)80019-4 9717856

[73] Vlachopoulos C., Ioakeimidis N., Miner M., Aggelis A., Pietri P., Terentes-Printzios D., et al. (2014). Testosterone deficiency: a determinant of aortic stiffness in men. Atherosclerosis, 233(1), 278–283. doi: 10.1016/j.atherosclerosis.2013.12.010 24529157

[74] Robison L.S., Gannon O.J., Salinero A.E., & Zuloaga K.L. (2019) Contributions of sex to cerebrovascular function and pathology. Brain Research, 1710, 43–60. doi: 10.1016/j.brainres.2018.12.030 30580011

[75] Matteis M., Troisi E., Monaldo B. C., Caltagirone C., & Silvestrini M. (1998a). Age and sex differences in cerebral hemodynamics: a transcranial Doppler study. Stroke; a Journal of Cerebral Circulation, 29(5), 963–967. doi: 10.1161/01.str.29.5.963 9596243

[76] Brackley K. J., Ramsay M. M., Broughton Pipkin F., & Rubin P. C. (1999). The effect of the menstrual cycle on human cerebral blood flow: studies using Doppler ultrasound. Ultrasound in Obstetrics & Gynecology: The Official Journal of the International Society of Ultrasound in Obstetrics and Gynecology, 14(1), 52–57. doi: 10.1046/j.1469-0705.1999.14010052.x 10461339

[77] Krejza J., Mariak Z., Huba M., Wolczynski S., & Lewko J. (2001). Effect of endogenous estrogen on blood flow through carotid arteries. Stroke; a Journal of Cerebral Circulation, 32(1), 30–36. doi: 10.1161/01.str.32.1.30 11136910

[78] Krejza J., Rudzinski W., Arkuszewski M., Onuoha O., & Melhem E. R. (2013). Cerebrovascular reactivity across the menstrual cycle in young healthy women. The Neuroradiology Journal, 26(4), 413–419. doi: 10.1177/197140091302600406 24007729 PMC4202816

[79] Krejza J., Siemkowicz J., Sawicka M., Szylak A., Kochanowicz J., Mariak Z., et al. (2003). Oscillations of cerebrovascular resistance throughout the menstrual cycle in healthy women. Ultrasound in Obstetrics & Gynecology: The Official Journal of the International Society of Ultrasound in Obstetrics and Gynecology, 22(6), 627–632. doi: 10.1002/uog.907 14689537

[80] Nevo O., Soustiel J. F., & Thaler I. (2007). Cerebral blood flow is increased during controlled ovarian stimulation. American Journal of Physiology. Heart and Circulatory Physiology, 293(6), H3265–H3269. doi: 10.1152/ajpheart.00633.2007 17965286

[81] Waldstein S. R., Rice S. C., Thayer J. F., Najjar S. S., Scuteri A., & Zonderman A. B. (2008). Pulse pressure and pulse wave velocity are related to cognitive decline in the Baltimore Longitudinal Study of Aging. Hypertension, 51(1), 99–104. doi: 10.1161/HYPERTENSIONAHA.107.093674 18025297

[82] Gorelick P. B., Nyenhuis D., American Society of Hypertension Writing Group, Materson B. J., Calhoun D. A., Elliott W. J., … Townsend R. R. (2012). Blood pressure and treatment of persons with hypertension as it relates to cognitive outcomes including executive function. Journal of the American Society of Hypertension: JASH, 6(5), 309–315 doi: 10.1016/j.jash.2012.08.004 22995799

[83] Lim S. L., Gao Q., Nyunt M. S. Z., Gong L., Lunaria J. B., Lim M. L., et al. (2016). Vascular Health Indices and Cognitive Domain Function: Singapore Longitudinal Ageing Studies. Journal of Alzheimer’s Disease: JAD, 50(1), 27–40. doi: 10.3233/JAD-150516 26639958

[84] Hajjar I., Goldstein F. C., Martin G. S., & Quyyumi A. A. (2016). Roles of Arterial Stiffness and Blood Pressure in Hypertension-Associated Cognitive Decline in Healthy Adults. Hypertension, 67(1), 171–175. doi: 10.1161/HYPERTENSIONAHA.115.06277 26527049 PMC4715367

[85] Suchy Y. (2015). Executive Functioning: A Comprehensive Guide for Clinical Practice. Oxford University Press.

[86] De la Torre J. C. (2002). Alzheimer disease as a vascular disorder: nosological evidence. Stroke; a Journal of Cerebral Circulation, 33(4), 1152–1162. doi: 10.1161/01.str.0000014421.15948.67 11935076

[87] Narkiewicz K., Kjeldsen S. E., & Hedner T. (2006). Hypertension and cardiovascular disease in women: Progress towards better understanding of gender‐specific differences? Blood Pressure, 15(2), 68–70. doi: 10.1080/08037050600750165 16754268

[88] Reich T., & Rusinek H. (1989). Cerebral cortical and white matter reactivity to carbon dioxide. Stroke; a Journal of Cerebral Circulation, 20(4), 453–457. doi: 10.1161/01.str.20.4.453 2494780

[89] Lu H., Xu F., Rodrigue K. M., Kennedy K. M., Cheng Y., Flicker B., et al. (2011). Alterations in cerebral metabolic rate and blood supply across the adult lifespan. Cerebral Cortex, 21(6), 1426–1434. doi: 10.1093/cercor/bhq224 21051551 PMC3097991

[90] Bhogal A. A., De Vis J. B., Siero J. C. W., Petersen E. T., Luijten P. R., Hendrikse J., et al. (2016). The BOLD cerebrovascular reactivity response to progressive hypercapnia in young and elderly. NeuroImage, 139, 94–102. doi: 10.1016/j.neuroimage.2016.06.010 27291492

[91] De Vis J. B., Hendrikse J., Bhogal A., Adams A., Kappelle L. J., & Petersen E. T. (2015). Age-related changes in brain hemodynamics; A calibrated MRI study. Human Brain Mapping, 36(10), 3973–3987. doi: 10.1002/hbm.22891 26177724 PMC6869092

[92] Desjardins M. (2015). Vascular correlates of aging in the brain: Evidence from imaging data. IRBM, 36(3), 158–165.

[93] Baraghis E., Bolduc V., Lefebvre J., Srinivasan V. J., Boudoux C., Thorin E., et al. (2011). Measurement of cerebral microvascular compliance in a model of atherosclerosis with optical coherence tomography. Biomedical Optics Express, 2(11), 3079–3093. doi: 10.1364/BOE.2.003079 22076269 PMC3207377

[94] Warnert E. A. H., Verbree J., Wise R. G., & van Osch M. J. P. (2016). Using High-Field Magnetic Resonance Imaging to Estimate Distensibility of the Middle Cerebral Artery. Neuro-Degenerative Diseases, 16(5–6), 407–410. doi: 10.1159/000446397 27449212 PMC5322578

[95] Golay X., & Petersen E. T. (2006). Arterial spin labeling: benefits and pitfalls of high magnetic field. Neuroimaging Clinics of North America, 16(2), 259–268, x. doi: 10.1016/j.nic.2006.02.003 16731365

[96] Halani S., Kwinta J. B., Golestani A. M., Khatamian Y. B., & Jean Chen J. (2015). Comparing cerebrovascular reactivity measured using BOLD and cerebral blood flow MRI: The effect of basal vascular tension on vasodilatory and vasoconstrictive reactivity. NeuroImage, Vol. 110, pp. 110–123. doi: 10.1016/j.neuroimage.2015.01.050 25655446 PMC5167565

[97] Alsop D. C., Detre J. A., Golay X., Günther M., Hendrikse J., Hernandez-Garcia L., et al. (2015). Recommended implementation of arterial spin-labeled perfusion MRI for clinical applications: A consensus of the ISMRM perfusion study group and the European consortium for ASL in dementia. Magnetic Resonance in Medicine: Official Journal of the Society of Magnetic Resonance in Medicine/Society of Magnetic Resonance in Medicine, 73(1), 102–116. 10.1002/mrm.25197 PMC4190138 24715426

