# Supplementary figures and images for: Sex moderations in the relationship between aortic stiffness, cognition, and cerebrovascular reactivity in healthy older adults
Source: PLoS One. 2021 Sep 28;16(9):e0257815. doi: 10.1371/journal.pone.0257815 (PMC8478243; doi:10.1371/journal.pone.0257815)

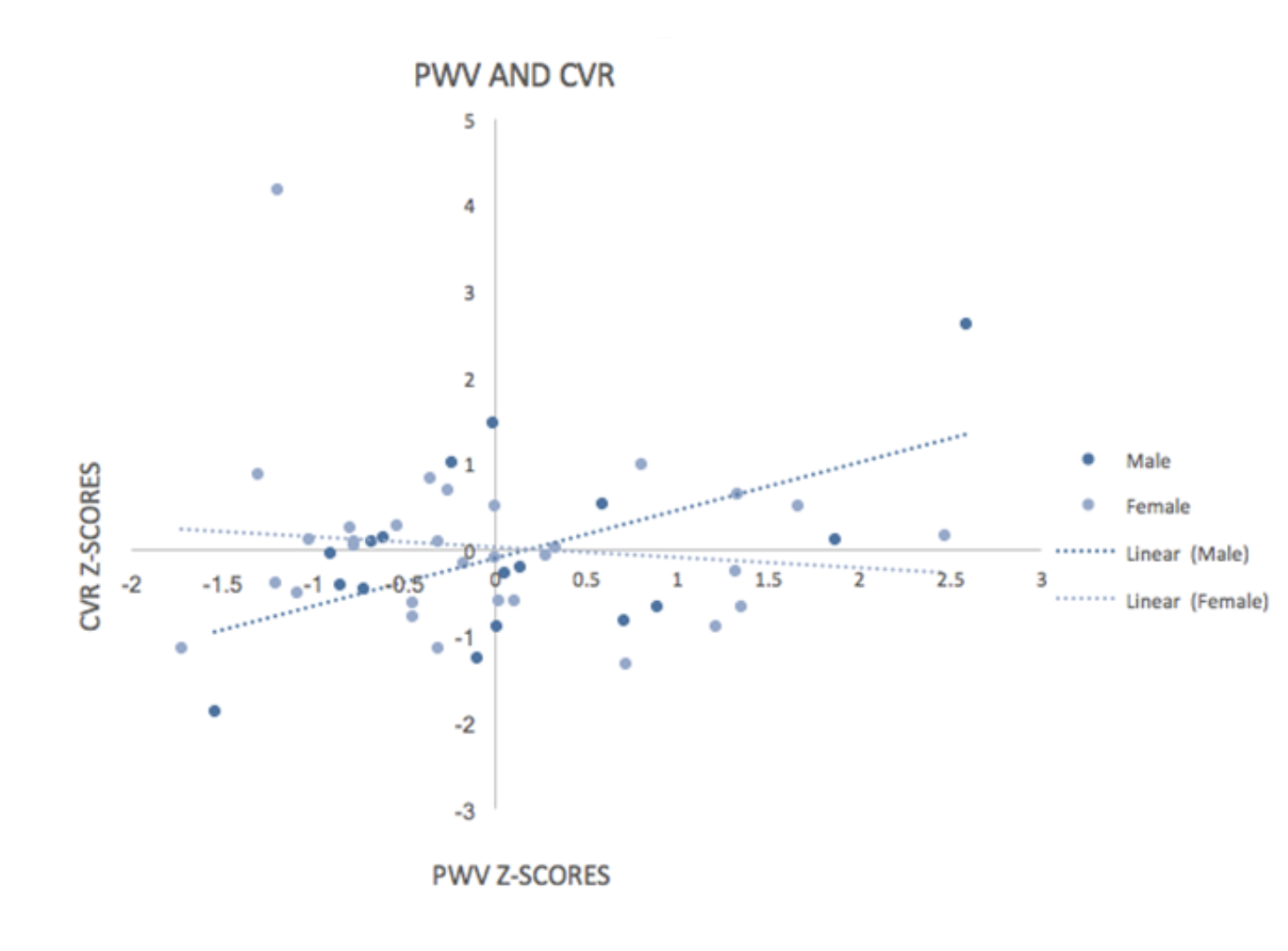

Supplement: S1 Fig — (TIF) [file pone.0257815.s001.tif]

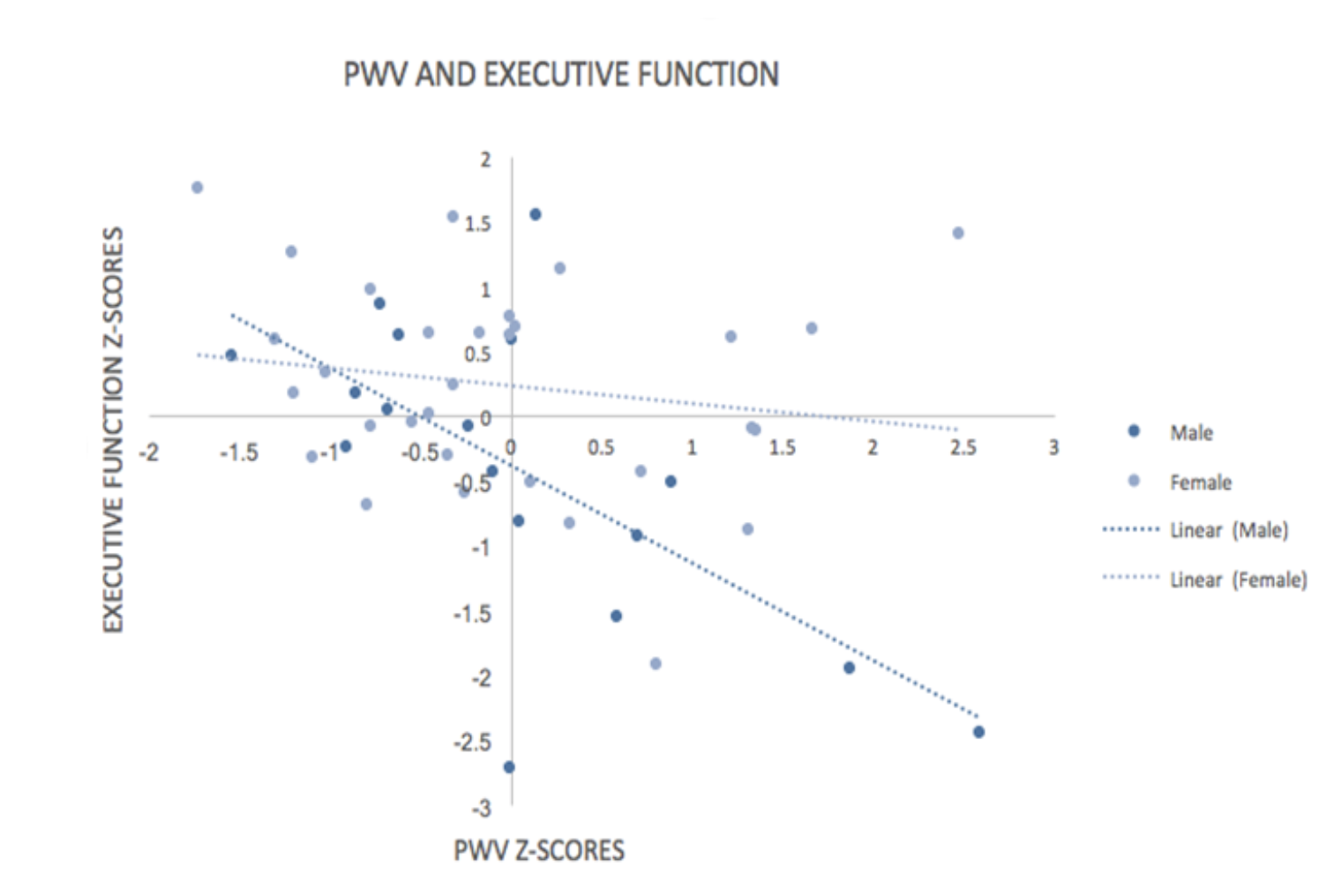

Supplement: S2 Fig — (TIF) [file pone.0257815.s002.tif]

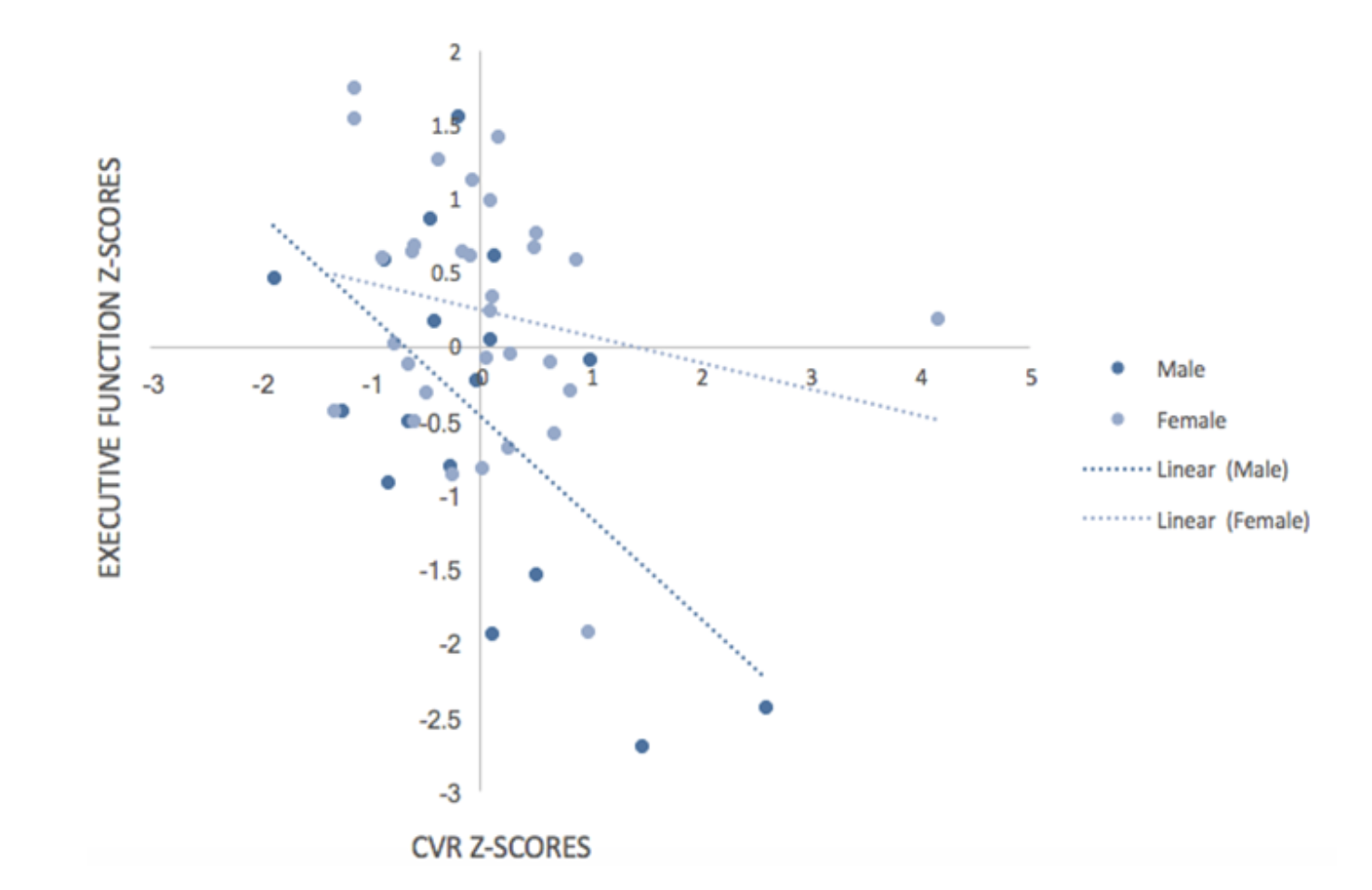

Supplement: S3 Fig — (TIF) [file pone.0257815.s003.tif]
